# Exercise adherence‐related perceptual responses to low‐load blood flow restriction resistance exercise in young adults: A pilot study

**DOI:** 10.14814/phy2.15122

**Published:** 2021-12-08

**Authors:** Tadashi Suga, Kento Dora, Ernest Mok, Takeshi Sugimoto, Keigo Tomoo, Shingo Takada, Takeshi Hashimoto, Tadao Isaka

**Affiliations:** ^1^ Faculty of Sport and Health Science Ritsumeikan University Kusatsu Shiga Japan; ^2^ Research Organization of Science and Technology Ritsumeikan University Kusatsu Shiga Japan; ^3^ Faculty of Lifelong Sport, Department of Sports Education Hokusho University Ebetsu Hokkaido Japan

**Keywords:** affect, enjoyment, perceived exertion, task motivation

## Abstract

Resistance exercise (RE) with blood flow restriction (BFR) is recognized as a beneficial strategy in increasing skeletal muscle mass and strength. However, the effects of BFR on changes in perceptual parameters, particularly those related to exercise adherence, induced by RE are not completely understood. In this study, we examined the exercise adherence‐related perceptual responses to low‐load BFR‐RE. Sixteen young males performed both BFR and non‐BFR (NBFR) sessions in a crossover design. The bilateral knee extensor low‐load RE was performed with a standard BFR‐RE protocol, consisting of four sets (total 75 repetitions), using 20% of one‐repetition maximum. BFR‐RE was performed with 200 mmHg pressure cuffs placed around the proximal region of the thighs. NBFR‐RE was performed without pressure cuffs. The ratings of perceived exertion and leg discomfort measured using the Borg's Scales were higher for BFR‐RE session than for NBFR‐RE session (both *p* < 0.001 for interaction effect). The Feeling Scale‐measured affect and Task Motivation Scale‐measured task motivation were lower for BFR‐RE session than for NBFR‐RE session (both *p* < 0.05 for interaction effect); by contrast, the Numerical Rating Scale‐measured perceived pain was higher for BFR‐RE session than for NBFR‐RE session (*p* < 0.001 for interaction effect). The Physical Activity Enjoyment Scale‐measured enjoyment immediately after RE was lower with BFR than with NBFR (*p* < 0.001). These findings suggest that BFR exacerbates the exercise adherence‐related perceptual responses to low‐load RE in young males. Therefore, further studies are needed to develop effective strategies that minimize the BFR‐RE‐induced negative effects on perceptual responses.

## Graphical abstract

1. This study examined the effects of blood flow restriction (BFR) on perceptual responses, including those related to exercise adherence, induced by low‐load resistance exercise (RE). 2. BFR exacerbates the exercise adherence‐related perceptual responses to low‐load RE. 3. Further studies are needed to develop effective strategies that minimize the BFR‐RE‐induced negative effects on perceptual responses.

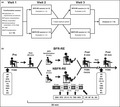

## 1 INTRODUCTION

Skeletal muscle weakness, presenting as decreased muscle mass and strength, is a prominent factor that indicates poor prognosis in older individuals and patients with chronic diseases (Ruiz et al., 2008). Long‐term intervention of resistance exercise (RE) results in numerous health improvements, including increased skeletal muscle mass and strength (American College of Sports Medicine, 2009; Williams et al., 2007). Generally, many guidelines have recommended the use of high‐loads for effective RE to certainly increase muscle mass and strength in healthy individuals (e.g., American College of Sports Medicine, 2009; Williams et al., 2007). However, the high‐load RE imposes considerable physical stresses in some individuals, especially older individuals and patients with chronic diseases, because of declining health of the cardiovascular and musculoskeletal systems (Williams et al., 2007). Furthermore, the high‐load RE also causes elevations in perceptual responses, including increased perceived exertion response and decreased affective response (Cavarretta et al., 2018), which can be considered barriers to exercise adherence (Trost et al., 2002). Therefore, novel RE method(s) with decreased exercise load and lowered perceptual responses that can provide training adaptations similar to those of high‐load RE would be useful in improving exercise adherence in various populations.

RE with blood flow restriction (BFR) is a unique method that uses low‐load (Patterson et al., 2019; Scott et al., 2015). The BFR‐RE results in muscle hypertrophy and strength gain more than non‐BFR (NBFR)‐RE in various populations (Lixandrão et al., 2018), including older individuals and patients with chronic diseases (Centner et al., 2019; Hughes et al., 2017). Moreover, muscle hypertrophy and strength gain induced by low‐load BFR‐RE is comparable to those induced by high‐load NBFR‐RE (Centner et al., 2019; Lixandrão et al., 2018). Furthermore, low‐load BFR‐RE can be performed more frequently than high‐load RE owing to lower muscle damage following RE (Dos Santos et al., 2020; Takarada et al., 2000); thus, BFR‐RE‐induced muscle adaptation can be obtained early within a short‐term (e.g., 2 weeks) because of increased training frequency throughout this period (Abe et al., 2005). Therefore, low‐load BFR‐RE is now recognized as a beneficial strategy to replace high‐load RE.

Despite many benefits of BFR‐RE, it exacerbates perceptual responses during the exercise (e.g., Bell et al., 2018; Loenneke, Kim, et al., 2015; Suga et al., 2009). Previous studies have reported that increases in perceived exertion parameters, such as the ratings of perceived exertion (RPE) and leg discomfort, during low‐load RE were greater with BFR than with NBFR (Bell et al., 2018; Suga et al., 2009). Furthermore, these perceived exertion responses during low‐load BFR‐RE were similar to or higher than those during high‐load RE (Bell et al., 2018; Loenneke, Kim, et al., 2015). Additionally, Silva et al. (2018) reported that mood states decreased after BFR‐RE, while they did not observe this after NBFR‐RE. These previous findings suggest that BFR may result in negative effects on perceptual responses to low‐load RE, which may contribute to decreasing exercise adherence (Cavarretta et al., 2018). However, limited perceptual parameters were measured in previous studies that examined the effect of BFR on perceptual responses to low‐load RE (Bell et al., 2018; Loenneke, Kim, et al., 2015; Silva et al., 2018; Suga et al., 2009). To the best of our knowledge, no study has examined the effects of BFR‐RE on major perceptual parameters related to exercise adherence (e.g., affect, task motivation, and enjoyment). In clinical settings, such information would be useful in creating effective protocols that would improve exercise adherence in various populations, especially older individuals and patients with chronic diseases.

Generally, changes in perceptual parameters induced by traditional exercise (i.e., exercise with NBFR) are dependent on changes in physiological parameters, including cardiovascular (e.g., heart rate (HR) and blood pressure), metabolic (e.g., blood lactate level), and neuromuscular (e.g., electromyographic (EMG) activity) parameters (Hampson et al., 2001). However, when the perceptual responses are greater in BFR‐RE than in NBFR‐RE, it is unclear whether these responses would be related to physiological responses. Additionally, although the perceptual responses to BFR‐RE are likely to be affected by the differences in body and lower limb sizes among subjects (Loenneke, Allen, et al., 2015; Loenneke, Kim, et al., 2015), it is poorly understood. In clinical settings, such information would also be useful in creating effective BFR‐RE protocols along with improving exercise adherence in various populations.

To clarify these practical questions, in this pilot study with young males, we first compared the responses in perceptual parameters, including exercise adherence‐related parameters, between low‐load knee extensor BFR‐ and NBFR‐REs. Second, we examined the relationship between perceptual and physiological responses to BFR‐ and NBFR‐REs. Third, we examined the relationships of physical characteristics, body composition, and anthropometrical parameters of the thigh with perceptual responses to BFR‐RE.

## 2 METHODS

### 2.1 Participants

To determine the sample size required for this study, we used the effect sizes (0.27–0.60) on two previous studies (Decker & Ekkekakis, 2017; Rose & Parfitt, 2007) that examined the changes in perceptual parameters (i.e., RPE and affect) induced by exercise, with a 2 (condition) × > 6 (time) two‐way repeated‐measures analysis of variance (ANOVA). The α‐ and β‐levels were set at 0.05 and 0.2 (80% power), respectively. The required minimum number of subjects was 6–16.

Sixteen young males (age: 20.9 ± 0.4 years, body height: 172.4 ± 1.2 cm, body mass: 61.2 ± 1.5 kg, body mass index: 20.6 ± 0.5 kg/m^2^) participated in this study; therefore, the number of subjects recruited in this study was sufficient for ensuring statistical power and sensitivity. The fasting blood glucose levels, and resting systolic blood pressure (SBP) and diastolic blood pressure (DBP) in the subjects were 95.5 ± 1.6 mg/dl, 112.1 ± 2.1 mmHg, 71.5 ± 1.2 mmHg, respectively, which were calculated as the mean values for each parameter obtained on experimental days 1 and 2. All the subjects were students studying sports and health sciences. The subjects had received the lecture(s) to practicing the measurements of one‐repetition maximum (1‐RM) and perceptual responses (e.g., RPE) during exercise, which were performed in this study. The subjects did not undergo any specific habitual physical training within the previous 3 years. However, many of them had participated in sports activity and/or exercise training for 2–3 h per week through the physical education lecture(s). Exclusion criteria for this study were as follows: (1) Athletes and trained individuals who engaged in specific sports and/or exercise training, because these candidates may exhibit different physiological responses induced by BFR‐RE compared to untrained individuals (Takada et al., 2012); (2) subjects who had a history of common orthopedic injuries and surgery of the tissues around the knee joints (e.g., including muscle, tendon, cartilage, and ligaments); (3) subjects with any known cardiovascular, pulmonary, and neurological disorders; (4) subjects who had symptoms of obesity (i.e., body mass index of ≥25.0 kg/m^2^), diabetes (i.e., fasting blood glucose of ≥126 mg/dl), and hypertension (i.e., SBP/DBP of ≥140/90 mmHg), which were based on the Japanese guidelines (Araki et al., 2020; Umemura et al., 2019). All participants were informed of the experimental procedures and potential risks and provided written consent to participate in this study. This study was approved by the Ethics Committee of Ritsumeikan University and conducted according to the Declaration of Helsinki.

### 2.2 Experimental design

**FIGURE 1:**
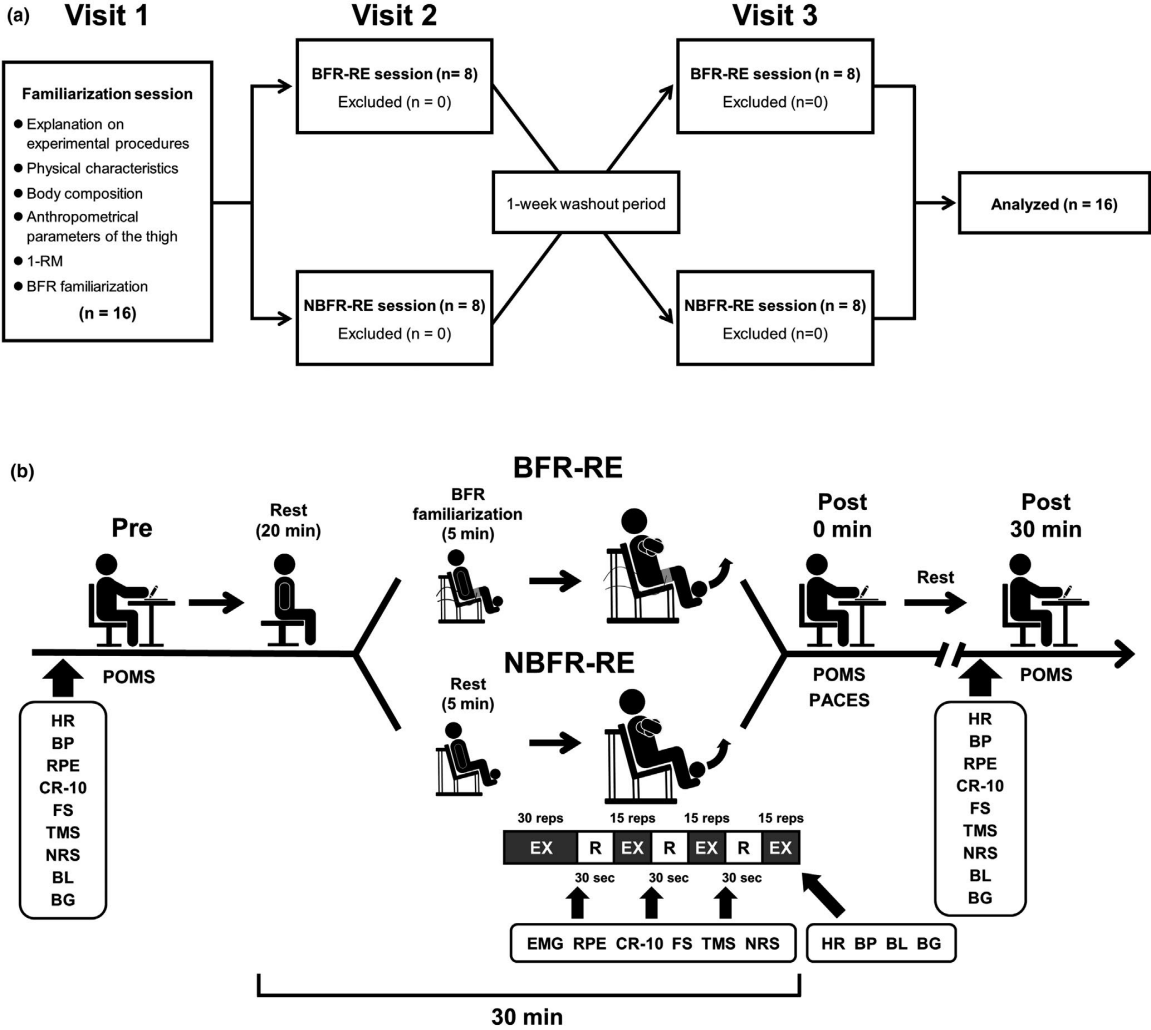
Experimental procedures of resistance exercise (RE) sessions with blood flow restriction (BFR) and non‐BFR (NBFR). Panel a shows the consort flow diagram for three visits of this study. In the familiarization day (i.e., visit 1), subjects received detailed explanations on experimental procedures (e.g., experimental protocols and perceptual parameters). Moreover, the subject's physical characteristics, body composition, anthropometrical parameters of the thigh, and one‐repetition maximum (1‐RM) of the bilateral knee extension were measured. Furthermore, the subjects were familiarized with the BFR maneuver. In the two experimental days (i.e., visits 2 and 3), the subjects completed both RE sessions with BFR and NBFR in a crossover design with a randomized and counterbalanced order. Panel b shows experimental procedures during the two experimental days. In each experimental day, the Borg's 15‐point Scale‐measured rating of perceived exertion (RPE), the Borg's Category‐Ratio 10‐point Scale (CR‐10)‐measured leg discomfort, the Feeling Scale (FS)‐measured affect, the Task Motivation Scale (TMS)‐measured task motivation, and the Numerical Rating Scale (NRS)‐measured perceived pain were collected throughout experimental session (i.e., before RE, during RE, and 30 min after RE). Cardiovascular (i.e., heart rate (HR) and blood pressure (BP)) and blood metabolite (i.e., blood lactate (BL) and blood glucose (BG)) parameters, and the Profile of Mood States (POMS)‐measured mood states were collected before RE, immediately after RE, and 30 min after RE. Electromyographic (EMG) activities of the three quadriceps femoris muscles were measured during every set of RE session. The Physical Activity Enjoyment Scale (PACES)‐measured enjoyment was collected immediately after RE

Experimental procedures of this study are presented in Figure 1. This study used a crossover design, whereby all subjects completed the two experimental RE sessions with BFR and NBFR, with a randomized and counterbalanced order. Each subject made a total of three visits to the laboratory over approximately 2 weeks. The two experimental sessions (i.e., second and third visits) were performed at approximately the same time (±1 h) in the morning, separated by a 1‐week period.

On the first visit, the subjects received detailed explanations on the experimental protocols and perceptual parameters. Thereafter, the subject's physical characteristics, body composition, anthropometrical parameters of the thigh, and 1‐RM of the bilateral knee extension were measured. After these measurements were completed, to minimize an excessive response to BFR, the subjects were familiarized with the BFR maneuver at sitting resting position using the familiarization method (see section Experimental conditions). Additionally, the subjects were instructed to avoid strenuous physical activity for 24 h before each of the two experiment days. The subjects were also instructed to abstain from food, caffeine, and alcohol for 12 h before each experiment day.

On the day of the experiments (i.e., second and third visits), the subjects performed either with low‐load BFR‐ or NBFR‐RE session on the leg extension machine (Life Fitness). Before the experiment on each day, the subjects again received detailed explanations on the perceptual parameters. Perceptual parameters (i.e., RPE, leg discomfort, affect, task motivation, and perceived pain) were measured throughout experimental session (i.e., before RE, during RE, and 30 min after RE). Cardiovascular (i.e., HR, SBP, DBP) and blood metabolite (i.e., blood lactate and glucose) parameters and mood states were measured before and immediately after RE, and 30 min after RE. Electromyographic (EMG) activities of the quadriceps femoris muscles were measured during every set of RE session. Enjoyment was measured immediately after RE.

### 2.3 Experimental conditions

The low‐load bilateral knee extensor RE was performed with a standard BFR‐RE protocol that involves 75 repetitions across four sets, consisting of 30 repetitions in the first set and 15 repetitions in each subsequent set, using a 20% of 1‐RM (Patterson et al., 2019; Scott et al., 2015). Rest interval lengths between sets were set at 30 s (Patterson et al., 2019; Scott et al., 2015). In the BFR‐RE, 8 cm wide tourniquet cuffs were wrapped around the proximal region of the thighs. The BFR pressure for BFR‐RE was set at 200 mmHg, as in previous studies (Fry et al., 2010; Fujita et al., 2007; Gundermann et al., 2012; Suga et al., 2009). To familiarize the subject with the BFR maneuver, the occlusion pressure was initially inflated at 100 mmHg for 30 s and then released for 10 s in sitting position on leg extension machine. Following the first BFR familiarization, the BFR pressure was gradually increased by 25 mmHg with 30‐s holding and 10‐s releasing. This BFR familiarization process was repeated until a final occlusion pressure at 200 mmHg was reached. Immediately before the BFR‐RE, the BFR was performed with the final occlusion pressure (i.e., 200 mmHg) and remained until the completion of exercise protocol. In the NBFR‐RE, the subjects performed a sitting rest with a same time (i.e., about 4–5 min) of the BFR familiarization on leg extension machine. After the sitting rest, the subjects performed same exercise protocol as the BFR‐RE, without the application of pressure cuffs.

### 2.4 1‐RM

On the familiarization visit, subject's 1‐RM was obtained by a successful concentric contraction of the bilateral knee extension to calculate the exercise load for low‐load RE, as previously described (e.g., Suga et al., 2009; Takada et al., 2012; Tsukamoto et al., 2017). The 1‐RM trial was designed using increments of 10 kg until 60%–80% of the perceived maximum is achieved. Then, the load was gradually increased by 1–5 kg weights until lift fail, in which the subject was not able to maintain proper form or to completely lift the weight. The last acceptable lift with the highest possible load was defined as 1‐RM. The mean 1‐RM of the bilateral knee extension in all subjects was 118 ± 4 kg. The mean load of 20% 1‐RM for both BFR‐ and NBFR‐REs in all subjects was 24 ± 1 kg.

### 2.5 Cardiovascular parameters

HR was measured continuously via telemetry (RS400; Polar Electro Japan). SBP and DBP were measured using a mercury manometer (FC‐110ST; Focal). Mean arterial pressure (MAP) was calculated as [(SBP − DBP)/3 + DBP].

### 2.6 Blood metabolites

Fingertip blood samples were collected to determine blood metabolite responses. Blood lactate and glucose levels were measured using lactate (Lactate Pro 2; Arkray) and glucose (Glutest Neo α; Sanwa Kagaku Kenkyusho) analyzers, respectively.

### 2.7 Quadriceps femoris EMG activity

The detailed method for measuring EMG activity of the quadriceps femoris has previously described (Tsukamoto et al., 2017). Peak EMGs of the rectus femoris, vastus lateralis, and vastus medialis muscles in the right leg during RE were calculated from the last five repetitions of all four sets. The peak EMG values in the five repetitions of each set for the three quadriceps femoris muscles were averaged and the mean EMG values were normalized to the EMG value measured during the knee extension maximal voluntary contraction.

### 2.8 RPE and leg discomfort

RPE was measured using the Borg's 15‐point Scale, which ranging from 6 (no exertion) to 20 (maximal exertion) (Borg, 1982). Rating of leg discomfort was measured using the Borg's Category‐Ratio 10‐point Scale (CR‐10), which ranges from 0 (nothing at all) to 10 (very, very strong) (Borg, 1982).

### 2.9 Affect

Affect was measured using the Feeling Scale (FS) (Hardy & Rejeski, 1989). The FS was an 11‐point bipolar scale, which ranges from −5 (very bad) to 5 (very good) with further descriptions at −3 (bad), −1 (fairly bad), 0 (neutral), 1 (fairly good), and 3 (good).

### 2.10 Task motivation

Task motivation was measured using the Task Motivation Scale (TMS) (Hutchinson et al., 2011). The TMS was an 11‐point scale, which ranges from 0 (nothing) to 10 (extremely strong) with further descriptions at 2 (weak), 5 (moderate), and 8 (strong).

### 2.11 Perceived pain

Perceived pain was measured using the Numerical Rating Scale (NRS) (Downie et al., 1978). The NRS was an 11‐point scale with descriptions at 0 (no pain at all), 5 (moderate pain), and 10 (worst pain imaginable).

### 2.12 Mood

Mood states were measured using a short version of the Profile of Mood States (POMS) (Shacham, 1983). This version was consisted of 35 questions and can be evaluated at six mood profiles: anger‐hostility, confusion‐bewilderment, depression‐dejection, fatigue‐inertia, tension‐anxiety, and vigor‐activity. Total mood disturbance (TMD) score was calculated based on methodology of the previous study (Shacham, 1983).

### 2.13 Enjoyment

Enjoyment was measured using the Physical Activity Enjoyment Scale (PACES) (Kendzierski & DeCarlo, 1991). The PACES was consisted of 18 questions, which is a total of 7 positive and 11 negative questions, with a 7‐point scale. The total score was used for analyses.

### 2.14 Physical characteristics, body composition, and anthropometrical parameters

Body height was measured using a stadiometer under barefoot condition. Body mass and whole‐body skeletal muscle and fat masses were measured using a bioelectrical impedance analysis with multiple impedance frequencies (InBody 720; Biospace) in barefoot condition and wearing only underwear, as in our previous study (Tottori et al., 2018). All anthropometrical parameters of the thigh were measured from the right leg. The thigh length was measured using a tape measure and defined as the distance between the lateral condyle of the femur and the greater trochanter. The thigh circumference was measured using a tape measure at 50% of the thigh length. The anterior and posterior muscle and subcutaneous fat thicknesses of the thigh were measured using a B‐mode US apparatus (SSD‐3500SV; Aloka) with a 7.5 MHz liner probe at a same location to the thigh circumference measurement (i.e., 50% of the thigh length).

### 2.15 Statistical analysis

All data were expressed as mean ± standard error of the mean. Changes in some perceptual parameters (i.e., RPE, leg discomfort, affect, task motivation, and perceived pain) throughout experimental session between BFR and NBFR conditions were analyzed using a 2 × 6 two‐way ANOVA. Changes in cardiovascular (i.e., HR and MAP) and blood metabolite parameters (i.e., blood glucose and lactate levels), and mood states throughout the two experimental sessions were analyzed using a 2 × 3 two‐way ANOVA. For all the ANOVAs, if the sphericity assumption was not met, Greenhouse–Geisser corrections were used. Specific differences were identified with a Bonferroni post‐hoc test or a paired *t*‐test. Comparisons of the mean values of the three quadriceps EMG activities during RE session between the two conditions were performed using a paired *t*‐test. Similar statistical analysis was used to compare enjoyment immediately after RE session between conditions. Partial eta squared (*η*_p_^2^) was calculated as the effect size to determine the magnitude of main effects of condition and time and interaction effect. Cohen's *d* was calculated as the effect size to determine the magnitude of difference in measured parameters between conditions (Cohen, 1992). Relationships between perceptual and physiological response to BFR‐ and NBFR‐REs were evaluated using a Pearson's product moment correlation coefficient. Similar statistical analyses were used to determine the relationships of physiological characteristics, body composition, and anthropometric parameters of the thigh with perpetual responses to BFR‐RE. The statistical significance level was defined at *p* < 0.05. All statistical analyses were conducted using IBM SPSS software (Ver. 19.0, IBM Corp).

## 3 RESULTS

### 3.1 Cardiovascular, blood metabolite, and quadriceps femoris EMG activity responses

**FIGURE 2:**
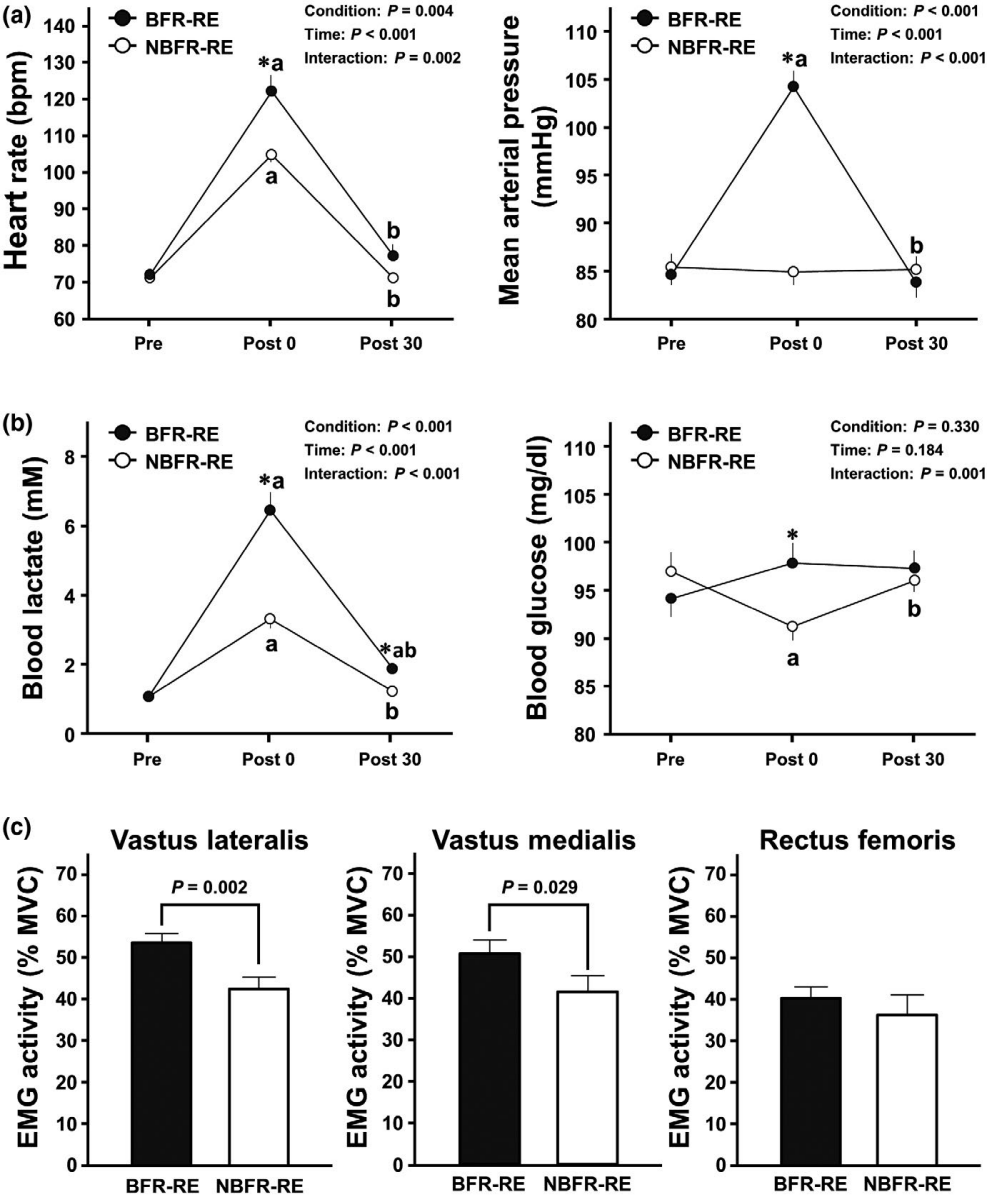
Cardiovascular, blood metabolite, and quadriceps femoris muscle activity responses to BFR‐ and NBFR‐REs. Panel a shows the changes in HR and BP throughout BFR‐ and NBFR‐RE sessions. Panel b shows the changes in BL and BG levels throughout BFR‐ and NBFR‐RE sessions. Panel c shows EMG activities of the three quadriceps femoris muscles during BFR‐ and NBFR‐REs. Values are presented as mean ± standard error of the mean. MVC; maximum voluntary contraction. *p < 0.05 versus NBFR‐RE, ^a^ *p* < 0.05 versus before RE (i.e., Pre), ^b^ *p *< 0.05 versus immediately after RE (i.e., Post 0)

Cardiovascular, blood metabolite, and quadriceps femoris EMG activity responses during BFR‐ and NBFR‐RE sessions are presented in Figure 2. Analyses of HR and MAP revealed significant main effects for condition and time and significant interaction effects (all *p* < 0.01, *η*_p_^2^ = 0.34–0.92). HR increased immediately after both BFR‐ and NBFR‐REs compared with that before REs (both *p* < 0.001, *d* = 3.85 and 4.73, respectively). MAP increased immediately after BFR‐RE but not NBFR‐RE compared with that before RE (*p* = 0.001, *d* = 3.45). HR and MAP immediately after RE was higher with BFR than with NBFR (both *p* ≤ 0.001, *d *= 1.26 and 3.05, respectively).

Blood lactate analysis revealed significant main effects for condition and time and a significant interaction effect (all *p* < 0.001, *η*_p_^2^ = 0.70–0.91). Blood lactate increased immediately after both BFR‐ and NBFR‐RE compared that before REs (both *p* < 0.001, *d* = 3.71 and 2.90, respectively). The increased blood lactate remained significant the 30‐min post‐exercise recovery period for BFR‐RE but not NBFR‐RE compared with that before RE (*p* < 0.001, *d* = 1.68). The blood lactate immediately after and 30 min after RE were higher with BFR than with NBFR (both *p *< 0.001, *d* = 1.91 and 1.27, respectively). Blood glucose analysis revealed a significant interaction effect (*p* = 0.001, *η*_p_^2^ = 0.38). The blood glucose decreased immediately after NBFR‐RE compared with that before RE (*p *= 0.010, *d* = 0.82). The blood glucose immediately after RE was higher with BFR than with NBFR (*p* = 0.010, *d* = 0.92).

Peak EMGs of the vastus lateralis and vastus medialis during RE were higher with BFR than with NBFR (both *p* < 0.05, *d* = 1.05 and 0.65, respectively).

### 3.2 RPE and leg discomfort responses

**FIGURE 3:**
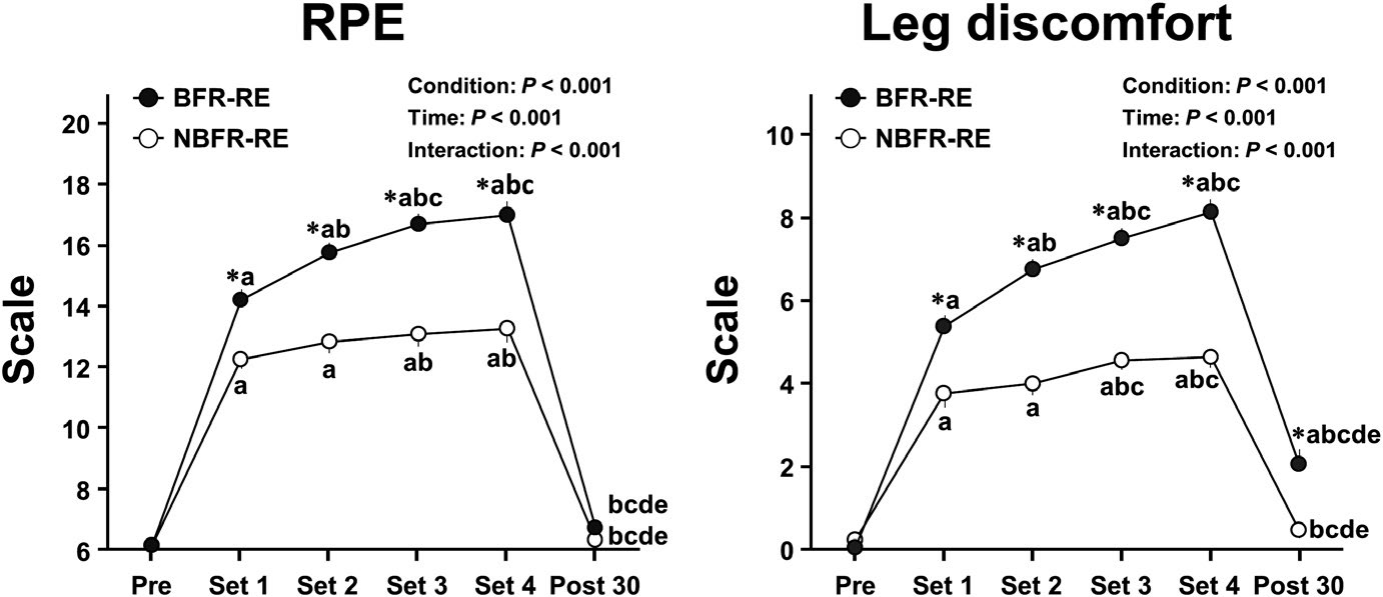
Changes in RPE and leg discomfort throughout BFR‐ and NBFR‐RE sessions. Values are presented as mean ± standard error of the mean. *p < 0.05 versus NBFR, ^a^ *p *< 0.05 versus Pre, ^b^ *p *< 0.05 versus  Set 1, ^c^ *p *< 0.05 versus  Set 2, ^d^ *p *< 0.05 versus  Set 3, ^e^ *p* < 0.05 versus Set 4 (i.e., Post 0).

Changes in RPE and leg discomfort throughout BFR‐ and NBFR‐RE sessions are shown in Figure 3. Analyses of RPE and leg discomfort revealed significant main effects for condition and time and significant interaction effects (all *p* < 0.001, *η*_p_^2^ = 0.53–0.98). RPE and leg discomfort increased during both BFR‐ and NBFR‐REs compared with those before REs (all *p* < 0.001, *d* = 3.25–10.84). The increased leg discomfort remained significant at the 30‐min post‐exercise recovery period for BFR‐RE but not NBFR‐RE compared with that before RE (*p* = 0.001, *d* = 2.06). The RPE and leg discomfort from the first to last sets during RE were higher with BFR than with NBFR (all *p *< 0.05, *d* = 1.33–3.04). Such a significant difference between conditions was remained for leg discomfort at the 30‐min post‐exercise recovery period (p <0.001, *d* = 1.41).

### 3.3 Affect, task motivation, and perceived pain responses

**FIGURE 4:**
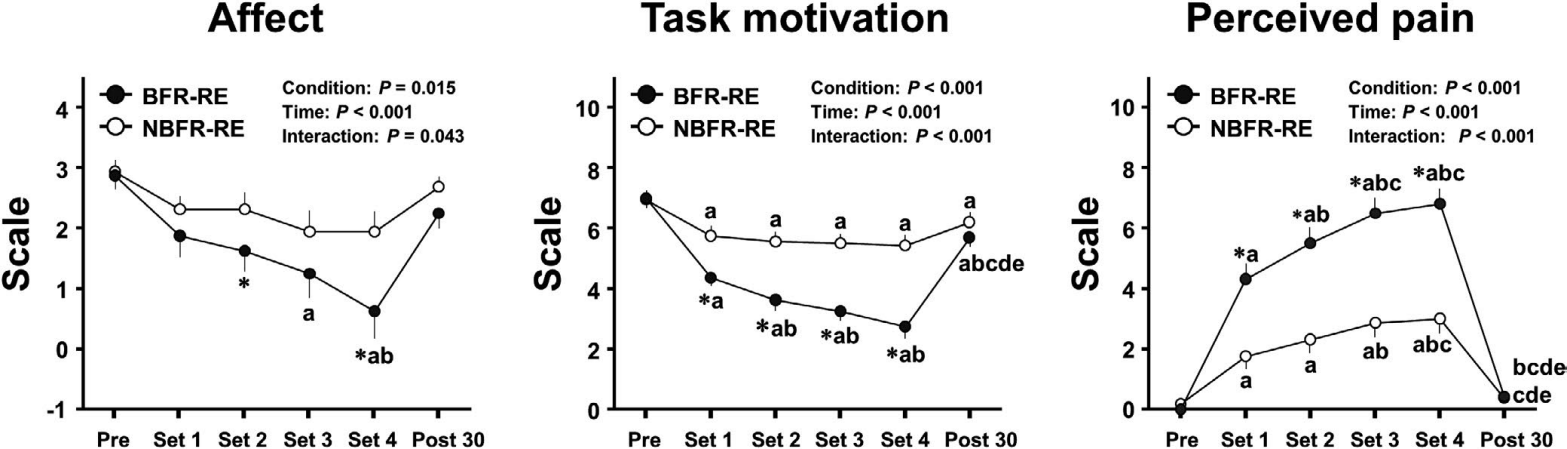
Changes in affect, task motivation, and perceived pain throughout BFR‐ and NBFR‐RE sessions. Values are presented as mean ± standard error of the mean. ^*^ *p* < 0.05 versus NBFR, ^a^ *p *< 0.05 versus Pre, ^b^ *p *< 0.05 versus  Set 1, ^c^ *p *< 0.05 versus  Set 2, ^d^ *p *< 0.05 versus  Set 3, ^e^ *p *< 0.05 versus Set 4 (i.e., Post 0).

Changes in perceptual psychological parameters throughout BFR‐ and NBFR‐REs sessions are presented in Figure 4. Analysis of affect, task motivation, and perceived pain revealed significant main effects for condition and time and significant interaction effects (all *p *< 0.05, *η*_p_^2^ = 0.17–0.85). Affect decreased at the third and last sets during BFR‐RE but not during NBFR‐RE compared with that before RE (both *p *< 0.05, *d* = 1.23 and 1.57, respectively). The affect at the second and last sets during RE were higher with BFR than with NBFR (all *p* = 0.05, *d* = 0.55 and 0.83, respectively). Task motivation decreased during both BFR‐ and NBFR‐REs compared with that before REs (all *p *< 0.05, *d* = 1.00–3.14). The decreased task motivation was remained significant at 30 min after both BFR‐ and NBFR‐REs compared with that before REs (both *p *< 0.05, *d* = 1.17 and 0.62, respectively). The task motivation from the first to last sets during RE were higher with BFR than with NBFR (all *p *< 0.001, *d* = 1.10–1.80). Perceived pain increased during both BFR‐ and NBFR‐REs compared with that before REs (all *p *< 0.05, *d* = 1.22–4.72). The perceived pain from the first to last sets during RE were higher with BFR than with NBFR (all *p *< 0.001, *d* = 1.36–1.90).

### 3.4 Mood responses

Changes in total mood disturbance and mood states throughout BFR and NBFR resistance exercise sessions are shown in Table 1. Total mood disturbance revealed main effect for time (*p* = 0.009, *η*_p_^2^ = 0.27). The total mood disturbance decreased 30 min after BFR‐RE compared with that immediately after RE (*p* = 0.048, *d* = 0.29). Among six mood states, confusion‐bewilderment analysis revealed a significant main effect for time (*p* = 0.014, *η*_p_^2^ = 0.25). The confusion‐bewilderment decreased 30 min after BFR‐RE compared with that before RE (*p* = 0.011, *d* = 0.29). Tension‐anxiety analysis revealed a significant main effect for time (*p* = 0.027, *η*_p_^2^ = 0.22). The tension‐anxiety decreased 30 min after NBFR‐RE compared with that before RE (*p* = 0.024, *d* = 0.25). Fatigue‐inertia analysis revealed a significant main effect for condition and time (both *p* < 0.05, *η*_p_^2^ = 0.32 and 0.48, respectively). The fatigue‐inertia increased immediately after both BFR‐ and NBFR‐REs compared with that before REs (both *p* =0.05, *d* = 1.13 and 0.63, respectively). The fatigue‐inertia decreased 30 min after both BFR‐ and NBFR‐REs compared with that immediately after REs (both *p *=0.05, *d* = 1.06 and 0.55, respectively). The fatigue‐inertia immediately after RE was higher with BFR than with NBFR (*p* = 0.008, *d* = 0.66).

**TABLE 1:** Changes in total mood disturbance and profile of mood states throughout resistance exercise (RE) sessions with blood flow restriction (BFR) and non‐BFR (NBFR)

	BFR‐RE			NBFR‐RE			*p values*		
	Pre	Post 0	Post 30	Pre	Post 0	Post 30	Condition	Time	Interaction
Total mood disturbance	4.4 ± 3.2	9.8 ± 3.3	3.8 ± 2.9^b^	4.2 ± 2.8	5.3 ± 3.2	2.8 ± 2.9	0.174	**0.009**	0.134
Anger‐hostility	1.6 ± 0.6	1.8 ± 0.9	1.0 ± 0.4	1.3 ± 0.4	1.1 ± 0.4	0.8 ± 0.4	0.383	0.171	0.352
Confusion‐bewilderment	4.1 ± 1.0	3.9 ± 0.9	3.0 ± 0.9^a^	3.9 ± 0.9	3.9 ± 1.1	3.1 ± 0.9	0.927	**0.014**	0.783
Depression‐dejection	1.3 ± 0.4	1.5 ± 0.4	1.2 ± 0.4	2.0 ± 0.6	1.4 ± 0.4	1.2 ± 0.4	0.444	0.167	0.115
Fatigue‐inertia	3.9 ± 0.9	7.6 ± 0.8^*a^	4.7 ± 0.6	3.4 ± 0.7	5.4 ± 0.9^a^	3.7 ± 0.7	**0.019**	**0.001**	0.140
Tension‐anxiety	3.9 ± 0.8	4.1 ± 0.9	3.3 ± 0.9	4.3 ± 0.9	3.6 ± 1.0	3.4 ± 0.9^a^	1.000	**0.027**	0.136
Vigor‐activity	10.4 ± 1.1	9.2 ± 1.4	9.4 ± 1.1	10.7 ± 0.9	10.2 ± 1.1	9.5 ± 0.9	0.393	0.101	0.546

Note

Values are presented as mean ± standard error of the mean. Bold *p* values indicate significant main effects of condition and time.

^*^ *p* < 0.05 versus NBFR.

^a^ *P* < 0.05 versus before RE (i.e., Pre).

^b^ *P* < 0.05 versus immediately after RE (i.e., Post 0).

### 3.5 Enjoyment response

**FIGURE 5:**
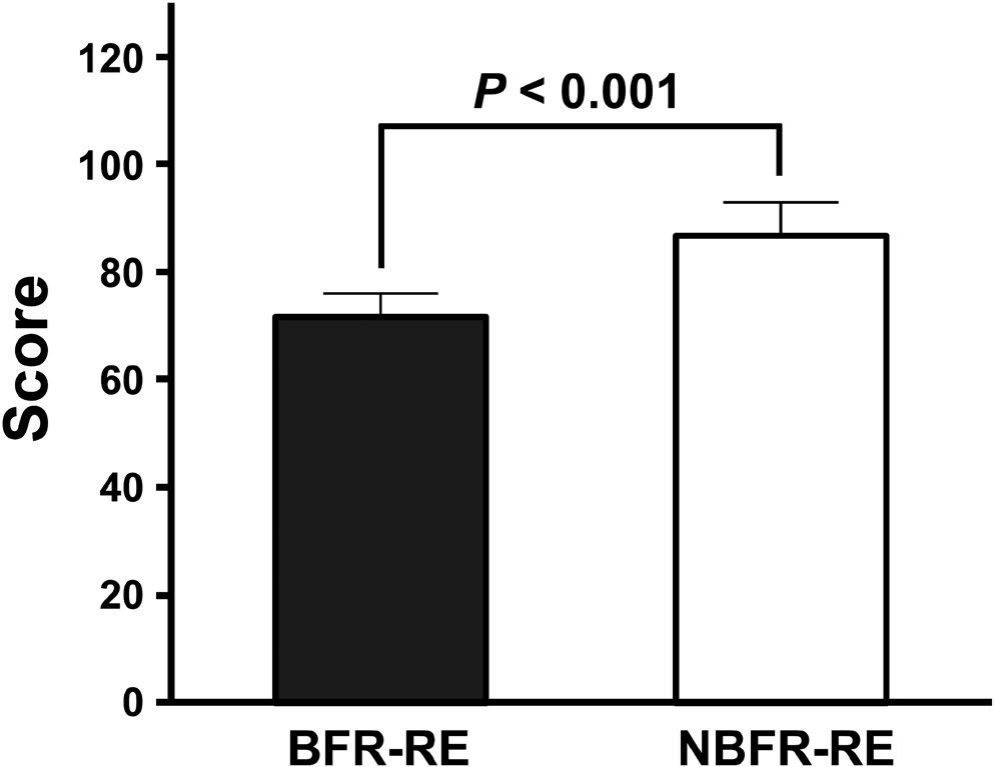
Comparison of enjoyment immediately after BFR‐ and NBFR‐REs. Values are presented as mean ± standard error of the mean. *Significant difference (*p* < 0.001) between BFR‐ and NBFR‐REs

Comparison of enjoyment immediately after BFR‐ and NBFR‐REs is presented in Figure 5. Enjoyment immediately after RE was lower with BFR than with NBFR (*p* < 0.001, *d* = 0.74).

### 3.6 Relationships of cardiovascular, blood metabolite, and quadriceps femoris EMG activity responses with perceptual responses to BFR‐ and NBFR‐REs

Correlation coefficients of cardiovascular, blood metabolite, and quadriceps femoris EMG activity responses with perceptual responses to BFR‐ and NBFR‐REs are summarized in Table 2. RPE response (i.e., ΔRPE), which evaluated as the difference between pre and post (i.e., at 5 set during each exercise) values, was correlated with HR, MAP, blood lactate and glucose responses, and vastus lateralis and rectus femoris EMG activities (*r* = 0.351–0.706, all *p* < 0.05). Leg discomfort response (i.e., ΔCR‐10) was correlated with HR, MAP, blood lactate and glucose responses, and vastus lateralis and vastus medialis EMG activities (*r* = 0.400–0.738, all *p* < 0.05). Affect response (i.e., ΔFS) was correlated with HR, MAP, blood lactate and glucose responses (*r* = −0.361 to −0.523, all *p* < 0.05). Perceived pain response (i.e., ΔNRS) was correlated with HR, MAP, blood lactate and glucose responses, and vastus lateralis EMG activity (*r* = 0.476–0.604, all *p* < 0.05). Task motivation response (i.e., ΔTMS) was correlated with HR, MAP, blood lactate and glucose responses, and vastus lateralis and vastus medialis EMG activities (*r* = −0.390 to −0.689, all *p* < 0.05). Total mood disturbance response (i.e., ΔTMD) was correlated with rectus femoris EMG (*r* = 0.482, *p* = 0.005). Enjoyment was correlated with MAP (*r* = −0.359, *p* = 0.043).

**TABLE 2:** Correlation coefficients of cardiovascular, blood metabolite, quadriceps femoris muscle activity responses with perceptual responses to BFR and NBFR‐REs

	ΔRPE	ΔCR−10	ΔFS	ΔNRS	ΔTMS	ΔTMD	PACES
ΔHR	**0.575 (*p* = 0.001)**	**0.551 (*p* = 0.001)**	**−0.459 (*p* = 0.008)**	**0.528 (*p* = 0.002)**	**−0.608 (*p* < 0.001)**	0.124 (*p* = 0.500)	−0.144 (*p* = 0.430)
ΔMAP	**0.706 (*p* < 0.001)**	**0.738 (*p *< 0.001)**	**−0.523 (*p* = 0.002)**	**0.602 (*p* < 0.001)**	**−0.689 (*p *< 0.001)**	0.278 (*p* = 0.124)	**−0.359 (*p *=* *0.043)**
ΔBlood lactate	**0.667 (*p* < 0.001)**	**0.637 (*p* < 0.001)**	**−0.397 (*p* = 0.024)**	**0.604 (p <0.001)**	**−0.616 (*p *< 0.001)**	0.174 (*p* = 0.341)	−0.116 (*p *= 0.526)
ΔBlood glucose	**0.598 (p <0.001)**	**0.460 (*p* = 0.008)**	**−0.361 (*p* = 0.042)**	**0.476 (*p* = 0.006)**	**−0.395 (*p* = 0.025)**	0.091 (*p* = 0.621)	−0.238 (*p* = 0.189)
Vastus lateralis iEMG	**0.534 (*p *=* *0.002)**	**0.571 (*p* = 0.001)**	−0.094 (*p* = 0.609)	**0.529 (*p* = 0.002)**	**−0.424 (*p* = 0.015)**	0.206 (*p* = 0.259)	−0.034 (*p* = 0.855)
Vastus medialis iEMG	0.301 (*p* = 0.094)	**0.400 (*p *=* *0.023)**	−0.058 (*p* = 0.751)	0.248 (*p* = 0.171)	**−0.390 (*p* = 0.027)**	0.160 (*p* = 0.383)	−0.139 (*p* = 0.447)
Rectus femoris iEMG	**0.351 (*p* = 0.049)**	0.230 (*p* = 0.206)	−0.200 (*p* = 0.273)	0.264 (*p* = 0.144)	–0.161 (*p* = 0.380)	**0.482 (*p* = 0.005)**	−0.162 (*p* = 0.376)

Note

*n* = 32 (16 × 2 conditions). Responses of perceptual and physiological parameters were defined as the differences (i.e., Δ) between pre and post (at 5 set during exercise) values during BFR‐ or NBFR‐RE sessions.

Bold values indicate significant correlations (p <0.05) between physiological and perceptual responses to BFR‐ and NBFR‐REs.

Abbreviations: CR‐10, Borg's Category‐Ratio 10‐point Scale (measuring leg discomfort); FS, Feeling Scale (measuring affect); iEMG, integrated electromyography; NRS, Numerical Rating Scale (measuring perceived pain); PACES, Physical Activity Enjoyment Scale (measuring enjoyment); RPE, Borg's rating of perceived exertion; TMD, Profile of Mood States‐measured total mood disturbance; TMS, Task Motivation Scale (measuring task motivation).

### 3.7 Relationships of physical characteristics, body composition, and anthropometrical parameters of the thigh with perceptual responses to BFR‐RE

Correlation coefficients of physical characteristics, body composition, and anthropometrical parameters of the thigh with perceptual responses to BFR‐RE are summarized in Table 3. Mean values of physical characteristics in the subjects were 172.4 ± 1.2 (range, 165.2–183.1) cm for body height, 61.2 ± 1.5 (range, 51.4–73.7) kg for body mass, and 20.6 ± 0.5 (range, 17.2–24.9) kg/m^2^ for body mass index. Mean values of body composition in the subjects were 13.9 ± 0.8 (range, 8.6–18.7) % for body fat percentage, 49.7 ± 1.1 (range, 43.3–57.1) kg for whole‐body skeletal muscle mass, and 8.6 ± 0.7 (range, 5.0 ± 13.0) kg for whole‐body fat mass. Mean values of anthropometrical parameters of the thigh in the subjects were 40.0 ± 0.4 (range, 38.0–43.0) cm for thigh length, 49.9 ± 0.8 (range, 45.4–55.3) cm for thigh circumference, 52.1 ± 1.6 (range, 38.4–62.3) mm for anterior thigh muscle thickness, 62.3 ± 1.2 (range, 52.8–70.9) mm for posterior thigh muscle thickness, 4.0 ± 0.5 (range, 1.2–6.9) mm for anterior thigh subcutaneous fat thickness, and 5.1 ± 0.6 (range, 1.9–10.5) mm for posterior thigh subcutaneous fat thickness.

**TABLE 3:** Correlation coefficients of physical characteristics, body composition, and anthropometric parameters of the thigh with perceptual responses to BFR‐RE

	ΔRPE	ΔCR−10	ΔFS	ΔNRS	ΔTMS	ΔTMD	PACES
Body height	0.168 (*p *= 0.534)	0.258 (*p *= 0.335)	0.022 (*p* = 0.935)	−0.437 (*p* = 0.091)	−0.168 (*p* = 0.533)	−0.321 (*p* = 0.225)	−0.305 (*p* = 0.251)
Body mass	−0.282 (*p *= 0.291)	−0.081 (*p *= 0.765)	0.448 (*p* = 0.082)	0.218 (*p* = 0.417)	**0.545 (*p* = 0.029)**	−0.375 (*p* = 0.152)	0.257 (*p* = 0.337)
Body mass index	−0.366 (*p *= 0.164)	−0.212 (*p *= 0.430)	0.437 (*p* = 0.090)	0.437 (*p* = 0.090)	**0.626 (*p* = 0.009)**	−0.205 (*p* = 0.446)	0.434 (*p* = 0.093)
Body fat percentage	−0.127 (*p *= 0.640)	−0.049(*p *= 0.856)	0.111 (*p* = 0.682)	0.204 (*p* = 0.449)	**0.523 (*p* = 0.038)**	−0.138 (*p* = 0.611)	0.283 (*p* = 0.287)
Whole body SKM	−0.251 (*p *= 0.348)	−0.057 (*p *= 0.833)	0.441 (*p* = 0.088)	0.123 (*p *= 0.649)	0.369 (*p *= 0.160)	−0.378 (*p* = 0.149)	0.129 (*p* = 0.634)
Whole body FM	−0.194 (*p *= 0.471)	−0.058 (*p *= 0.832)	0.258 (*p* = 0.335)	0.238 (*p* = 0.374)	**0.603 (*p* = 0.013)**	−0.219 (*p* = 0.416)	0.323 (*p* = 0.222)
Thigh length	0.185 (*p *= 0.493)	0.285 (*p *= 0.284)	0.181 (*p* = 0.502)	**0.561 (*p* = 0.024)**	−0.058 (*p *= 0.830)	−0.387 (*p* = 0.139)	−0.162 (*p* = 0.548)
Thigh circumference	**−0.517 (*p *=* *0.040)**	−0.304 (*p *= 0.252)	0.427 (*p* = 0.099)	0.331 (*p* = 0.211)	**0.635 (*p *=* *0.008)**	−0.204 (*p* = 0.447)	0.338 (*p* = 0.201)
Anterior thigh MT	−0.316 (*p *= 0.233)	−0.231 (*p *= 0.389)	0.186 (*p* = 0.491)	0.094 (*p* = 0.730)	0.428 (*p* = 0.098)	−0.249 (*p* = 0.352)	0.227 (*p* = 0.399)
Posterior thigh MT	−0.298 (*p *= 0.263)	−0.211 (*p *= 0.432)	0.250 (*p* = 0.351)	0.385 (*p* = 0.141)	**0.559 (*p *=* *0.024)**	−0.245 (*p* = 0.361)	0.469 (*p* = 0.067)
Anterior thigh SFT	−0.238 (*p *= 0.375)	−0.072 (*p *= 0.792)	−0.076 (*p* = 0.780)	0.159 (*p* = 0.557)	**0.602 (*p* = 0.014)**	−0.228 (*p *= 0.395)	0.088 (*p* = 0.747)
Posterior thigh SFT	0.019 (*p *= 0.946)	0.075 (*p *= 0.783)	0.043 (*p* = 0.874)	0.104 (*p* = 0.701)	0.275 (*p *= 0.303)	−0.232 (*p* = 0.386)	0.221 (*p* = 0.410)

Note

*n* = 16. Bold values indicate significant correlations (p = 0.05) of physical characteristics, body composition, and anthropometric parameters of the thigh with perceptual responses to BFR‐RE.

Abbreviations: FM, fat mass; MT, muscle thickness; SFT, subcutaneous fat thickness; SKM, skeletal muscle mass.

RPE response was correlated with the thigh circumference (*r* = −0.517, *p* = 0.040). Perceived pain response was correlated with the thigh length (*r* = −0.561, *p* = 0.024). Task motivation response was correlated with the body mass, body mass index, body fat percentage, whole‐body fat mass, thigh circumference, posterior thigh muscle thickness, and anterior thigh subcutaneous fat thickness (*r* = 0.523–0.635, all *p* < 0.05).

## 4 DISCUSSION

We and others have previously reported that increases in RPE and leg discomfort assessed using the Borg's 15‐point and CR‐10 Scales during low‐load RE were greater with BFR than with NBFR (Bell et al., 2018; Suga et al., 2009). In the present study, we also determined greater RPE and leg discomfort responses during BFR‐RE than those during NBFR‐RE; thus, the present findings corroborate the results of previous studies (Bell et al., 2018; Suga et al., 2009). Additionally, Silva et al. (2019) reported that leg discomfort at 30 min after low‐intensity aerobic exercise (i.e., slow running or fast walking) was higher for BFR than for NBFR; however, to the best of our knowledge, no study has examined the prolonged effect of the increases in RPE and leg discomfort induced by low‐load BFR‐RE. In the present study, we determined that leg discomfort, but not RPE, was higher 30 min after BFR‐RE than that before RE, whereas no such effect was observed 30 min after NBFR‐RE; further, the leg discomfort at 30 min after RE was higher for BFR than for NBFR. Therefore, this present finding suggests that BFR‐induced negative response for leg discomfort may persist for at least 30 min during the post‐exercise recovery period.

Cavarretta et al. (2018) reported that the FS‐measured affect is increased by traditional low‐ and moderate‐intensity RE protocols. In contrast, Portugal et al. (2015) reported that although affect did not change during RE protocols with low‐ (40% 1‐RM) or moderate‐ (60% 1‐RM) load, it decreased during a high‐load (80% 1‐RM) RE protocol. Elsangedy et al. (2018) also reported that RE‐induced decrease in affect was parallel to an increase from low to high exercise loads. Therefore, affective response to RE appears to be dependent on exercise loads, particularly in a range of moderate to high loads. However, no study has examined the effect of BFR on affective responses during RE. In the present study, affect decreased during BFR‐RE but not NBFR‐RE compared with that before RE, and this change was greater during BFR‐RE than during NBFR‐RE. This present finding suggests that, despite a use of low‐load, BFR may result in negative effect on affective responses to low‐load RE.

When aerobic exercise was performed, Brown et al. (2016) reported no change in the TMS‐measured task motivation during high‐intensity interval exercise. In contrast, Stork et al. (2015) reported that task motivation decreased during sprint interval exercise. Thus, vigorous aerobic exercise may result in a decrease in task motivation. On the other hand, no study has examined the effect of RE on task motivation. In the present study, although task motivation decreased during both BFR‐ and NBFR‐REs compared with that before each RE, this change was greater during BFR‐RE than during NBFR‐RE. This present finding suggests that, similar to affect, task motivation during low‐load RE may result in a more negative response with BFR than with NBFR.

Prior to this study, the effect of BFR‐RE on the NRS‐measured perceived pain was unknown. In the present study, although perceived pain increased during both BFR‐ and NBFR‐REs compared with that before each RE, this change was greater during BFR‐RE than during NBFR‐RE. Sharma et al. (2014) reported that despite being at rest, BFR increased perceived pain, potentially due to mechanical pain related to the imposed BFR pressure. The potential relationships may exist between an increase in pain and excessive other perceptual responses (Bennell et al., 2014); therefore, the BFR‐induced increase in perceived pain may be the basis for the negative responses of other measured perceptual parameters during low‐load RE.

The POMS‐measured mood states, including TMD, is negatively changed by RE in a dose‐dependent manner (Chan et al., 2019). Furthermore, Silva et al. (2018) reported that TMD measured using the Brunel Mood Scale showed a negative response immediately after BFR‐RE compared with that before exercise; however, they did not compare the changes in TMD between BFR‐ and NBFR‐REs. Another study by Silva et al. (2019) also reported that the Brunel Mood Scale‐measured TMD immediately after low‐intensity aerobic exercise was negatively changed by imposing BFR but not NBFR compared with that before exercise; further, the level of negative response induced by the low‐intensity aerobic exercise with BFR was similar to that induced by high‐intensity aerobic exercise. In the present study, the POMS‐measured TMD was not significantly changed by both BFR‐ and NBFR‐REs. Nevertheless, the TMD showed a significant difference between immediately after and 30 min after BFR‐RE but not NBFR‐RE. Additionally, although fatigue‐inertia increased immediately after both BFR‐ and NBFR‐REs compared with that before each RE, this response was higher immediately after BFR‐RE than immediately after NBFR‐RE. This present finding suggests that BFR‐RE may slightly result in negative mood states more than NBFR‐RE.

Enjoyment can be considered an important perceptual parameter related to exercise adherence (Decker & Ekkekakis, 2017; Kendzierski & DeCarlo, 1991; Trost et al., 2002). Nevertheless, only few studies have examined the effect of RE on the PACES‐measured enjoyment (Greene & Petruzzello, 2015; Richardson et al., 2018). Greene and Petruzzello (2015) reported that enjoyment immediately after RE was lower with a high load (100% of 10‐RM) than with a moderate load (70% of 10‐RM). In contrast, Richardson et al. (2018) reported that enjoyment was similar between low‐ and high‐load REs when the work volume was matched. In the present study, despite the use of a same work volume, enjoyment was lower immediately after BFR‐RE than immediately after NBFR‐RE. This present finding suggests that BFR‐RE may have a large barrier to exercise adherence of some individuals owing to the RE‐induced negative response of enjoyment, as well as other measured perceptual parameters.

Changes in perceptual parameters induced by RE can be associated with physiological responses, such as cardiovascular, metabolic, and neuromuscular responses (Hampson et al., 2001). In the present study, cardiovascular (i.e., HR and MAP), blood metabolite (i.e., blood lactate and glucose), and neuromuscular (i.e., quadriceps femoris EMGs) responses during RE were higher with BFR than with NBFR. Furthermore these physiological responses were correlated with perceptual responses to BFR‐ and NBFR‐REs. Additionally, we have previously reported that changes in intramuscular metabolites (e.g., creatine phosphate depletion, increased inorganic phosphate, and decreased intracellular pH) during RE was greater with BFR than with NBFR, and that these intramuscular metabolic responses were concordant with an increase in leg discomfort (Suga et al., 2009). The increases in the peripheral and systemic physiological responses induced by BFR‐RE may enhance central sensitization (Craig, 2002), potentially by activating the central neural system, including the sympathetic nervous system (Spranger et al., 2015). In the present study, blood glucose level immediately after RE was higher with BFR than with NBFR, which can be partially explained by the BFR‐induced sympathetic nervous system activation, because of the close relationship between blood glucose response and sympathetic nervous system activation during exercise (Nonogaki, 2000). Therefore, the BFR‐induced negative responses on perceptual parameters to low‐load RE may be at least partially due to greater physiological responses during BFR‐RE than during NBFR‐RE.

In this study, we observed that physical characteristics (i.e., body mass and body mass index), body composition (i.e., body fat percentage and whole‐body fat mass), and anthropometrical parameters of the thigh (i.e., length, circumference, posterior muscle thickness, and anterior subcutaneous fat thicknesses) were correlated with some responses of measured perceptual parameters to BFR‐RE. Based on these correlations, it could be surmised that subjects with smaller body and lower limb sizes may induce greater negative effects of perceptual responses during BFR‐RE than those with larger body and lower limb sizes. Therefore, in the clinical settings, physical characteristics, body composition, and anthropometrical parameters of the thigh may help predict the negative effects of BFR on perceptual responses to low‐load RE.

A major limitation of this study is that, although recent guidelines recommend the use of the relative BFR pressure for performing BFR‐RE based on the subject's arterial occlusion pressure (Patterson et al., 2019; Scott et al., 2015), we employed an absolute BFR pressure of 200 mmHg for performing BFR‐RE uniformly among all subjects; thus, the BFR‐RE in this study may have been performed with relatively different BFR pressures among the subjects. The within‐subject difference in the relative BFR pressures for performing BFR‐RE affects the degree of the exercise‐induced perceptual responses (Bell et al., 2018). Hence, the use of an absolute BFR pressure employed in the present study might lead to an inconsistent evaluation of the perceptual responses to BFR‐RE among the subjects. Furthermore, this might affect the correlations between physiological and perceptual responses to BFR‐ and NBFR‐REs and the correlations of physical characteristics, body composition, and anthropometrical parameters of the thigh with perceptual responses to BFR‐RE. To clarify the findings of the present study, using the relative BFR pressure based on the subject's arterial occlusion pressure, further studies are needed to reexamine the effects of BFR on perceptual responses to low‐load RE.

Another limitation of this study is that we recruited only young males; therefore, it is unclear whether the present findings can be generalized to other populations. In particular, athletes and trained individuals may exhibit different BFR‐RE‐induced physiological responses compared to untrained individuals (Takada et al., 2012). Furthermore, applications of BFR exercises, including RE, to increase skeletal muscle mass and strength may be more useful in older individuals and patients with chronic diseases than in young individuals; this is because the BFR‐RE is being recognized as a beneficial strategy in these populations (Centner et al., 2019; Hughes et al., 2017), including older patients with congestive heart failure (Groennebaek et al., 2019). To extend the findings of this pilot study involving young males, further studies are needed to examine the effects of BFR on perceptual responses to low‐load RE in various populations and identify an effective strategy that minimize the low‐load BFR‐RE‐induced negative effects on perceptual response, while also taking into consideration the uniqueness of each population.

In a practical application from the findings of this study, the correlations of physical characteristics, body composition, and anthropometrical parameters of the thigh with perceptual responses to BFR‐RE may help estimate the relative BFR pressure that equalizes the differences in perceptual responses to BFR exercise among the subjects. It is recommended that the relative BFR pressure is estimated based on the subject's arterial occlusion pressure (Patterson et al., 2019; Scott et al., 2015). Nevertheless, the measurement of the arterial occlusion pressure is generally done for using the Doppler ultrasound. Although many research institutes have ultrasonographic devise, in routine clinical setting, few facilities have this devise. In contrast, simple measurements, such as physical characteristics (i.e., body mass and body mass index) and thigh circumference, could be measured at such facilities. Loenneke, Kim, et al. (2015) used the relative BFR pressure based on the subject's thigh circumference for performing low‐load knee extensor BFR‐RE, which is because of the correlation between the limb circumference and arterial occlusion pressure (Loenneke, Allen, et al., 2015). Therefore, physical characteristics, body composition, and anthropometrical parameters of the thigh, particularly the thigh circumference, may be useful parameters to apply the optimal BFR pressure for performing BFR exercise in the clinical settings, which can be used as surrogates for measuring the arterial occlusion pressure.

## 5 CONCLUSION

This study demonstrated that perceptual responses, including those related to exercise adherence, to low‐load knee extensor RE were greater with BFR than with NBFR. The present findings suggest that BFR may have negative effects on perceptual responses to the low‐load RE, which can be considered barriers to exercise adherence for some individuals. To further popularize the BFR‐RE in the clinical settings, there is needed to develop effective strategies that minimize the BFR‐induced negative effects on perceptual response.

## CONFLICT OF INTEREST

The authors declare that they have no conflict of interest.

## AUTHOR CONTRIBUTIONS

TadaS conceived and designed the experiment; TadaS, KD, EM, TakeS, and KT performed the experiments; TadaS and KD analyzed the data; TadaS, KD, EM, TakeS, KT, ST, TH, and TI interpreted the results of the experiments; TadaS and KD wrote the manuscript; TadaS, TS, TH, and TI edited and revised the manuscript. All authors have read and approved the manuscript.
